# Melatonin Supplementation During Middle Gestation Alleviates Intrauterine Growth Restriction in Piglets and Is Associated with Alterations in Placental DNA Methylation

**DOI:** 10.3390/ijms27209046

**Published:** 2026-10-12

**Authors:** Likai Wang, Huigang Han, Zhenhua Xue, Laiqing Yan, Zixia Shen, Jiarui Gao, Pengyun Ji, Bingyuan Wang, Guoshi Liu

**Affiliations:** 1 National Engineering Laboratory for Animal Breeding, Key Laboratory of Animal Genetics and Breeding of the Ministry of Agricultural, State Key Laboratory of Animal Biotech Breeding, Frontiers Science Center for Molecular Design Breeding, College of Animal Science and Technology, China Agricultural University, Beijing 100193, China; wanglikai@sxu.edu.cn (L.W.); 15550013237@163.com (H.H.); xue1928@163.com (Z.X.); laiqingyan@cau.edu.cn (L.Y.); shenzixiaa@163.com (Z.S.); gaojiarui98@126.com (J.G.); jipengyun@cau.edu.cn (P.J.); wangbingyuan@cau.edu.cn (B.W.); 2 Institutes of Biomedical Sciences, College of Life Science, Shanxi University, Taiyuan 030006, China; 3 College of Animal Science, Xinjiang Agricultural University, Urumqi 830052, China

**Keywords:** performance, melatonin, methylation, sows

## Abstract

Intrauterine growth restriction (IUGR) has a negative impact on reproductive performance in domestic animals. In this study, we attempt to explore the etiology of IUGR in sows and test the potential beneficial effects and underlying molecular mechanisms of melatonin on IUGR. The results indicated that there was a negative correlation between the concentration of melatonin in the peripheral blood of pregnant sows and the ratio of IUGR, and the concentration of melatonin in the umbilical cord blood of IUGR piglets was abnormally decreased. Supplementing sows with an appropriate dose of melatonin can increase the average birth weight of piglets and reduce the proportion of IUGR piglets. Reduced representation bisulfite sequencing revealed a direct association between abnormal DNA methylation in the placenta of sows and IUGR piglets. The increased DNA methylations of key genes responsible for placenta functions downregulated their expressions. Gene downregulation caused placenta dysfunction and IUGR. Melatonin significantly reduced DNA methylation of genes related to placental function. We identified that melatonin supplementation during mid-gestation stages specifically targeted the CGI, CGI shore, exon, intron, and 3′ untranslated regions of placental genes. The results provide novel information to target the high incidence of IUGR in pigs as well as other species.

## 1. Introduction

Intrauterine growth restriction (IUGR) generally refers to a phenomenon where the growth rate of the fetus within the uterus is below the normal level; i.e., the fetal weight is, at least, 10% lower than that of a normal fetus of the same gestational stage. This condition not only affects the health of the fetus and newborn but also impacts the well-being of the adult, including increased susceptibility to metabolic syndrome, cardiovascular diseases, and type 2 diabetes mellitus [1]. As a polytocous domestic animal, pigs are susceptible to severe intrauterine growth restriction, which could affect up to 25% of the piglets in each litter [2,3,4]. Piglets affected by IUGR often exhibit reduced survival rates, impaired growth performance, and poor meat quality [5,6]. The etiology of IUGR is multifold; one factor is the placenta, which plays a crucial role. The placenta functions as the interface for nutrient and waste exchange between maternal and fetal circulations, and its dysfunction inevitably results in IUGR [7]. Such disturbances in placental transport may compromise the development and function of vital fetal organs and tissues, including the brain, heart, intestines, and skeletal muscles [8,9].

To detect IUGR early and fully understand its relation to the placenta, it is necessary to identify the molecular markers of this disorder in pigs. Alterations in DNA methylation in IUGR placentas have been widely investigated as potential molecular markers of IUGR. Due to the variations in gene expression among different placental cell types, as well as alterations in the placental cell composition throughout gestation, the DNA methylation profiles of placental tissue undergo continuous changes throughout the pregnancy [10,11]. DNA methylation crucially directs the gene expression patterns related to placental formation and function [12,13,14]. For example, DNA methylation is involved in the regulation of trophoblast invasion and placental development [15,16]. Supplementation with choline, a methyl donor, during pregnancy has been shown to modulate placental inflammation and angiogenesis in mice [17]. The evidence strongly supports that gene methylation in the placenta during pregnancy is closely related to fetal growth and development.

In addition, increased production of reactive oxygen species (ROS) and decreased antioxidant capacity during pregnancy can contribute to the development of IUGR through oxidative stress [18]. Therefore, maintaining the balance between ROS production and antioxidant defenses may help reduce the risk of IUGR. Melatonin is a naturally occurring molecule with potent antioxidant and anti-inflammatory properties [19,20]. It has been found that the serum melatonin level in pregnant women is higher than that of their non-pregnant counterparts, suggesting that the increased melatonin level in pregnant women is probably from the placenta [21,22]. In contrast, in cases of IUGR caused by placental dysfunction, the melatonin level in peripheral blood is significantly reduced, and the expressions of gene melatonin receptors, melatonin receptor 1A (MTNR1A) and melatonin receptor 1B (MTNR1B), are also downregulated [23,24]. Melatonin supplementation from gestational days 90 to 114 in sows has been shown to significantly increase piglet birth weight, weaning survival rate, and body weight compared with those in a control group [25]. These findings suggest that melatonin may play an important role in maintaining placental function and supporting fetal development. In addition to its anti-oxidative and anti-inflammatory action, the beneficial effects of melatonin on IUGR may also be attributed to its action to modify DNA methylation during pregnancy. Melatonin can reprogram DNA methylation to stimulate cell differentiation and to promote the development of cloned embryos [26,27]. Additionally, melatonin exerts a protective effect against LPS-induced abnormal pregnancy in mice through MTNR1B-m6A [28]. Furthermore, differences in DNA methylation patterns have been observed between transgenic dairy goats overexpressing acetylserotonin O-methyltransferase (ASMT), a rate-limiting enzyme involved in melatonin synthesis, and their wild-type counterparts [29]. Collectively, these findings suggest that melatonin may participate in epigenetic regulation, including the modulation of DNA methylation. However, whether melatonin improves placental function by regulating placental DNA methylation remains unclear.

In this study, we hypothesized that melatonin supplementation during gestation would be associated with alterations in placental DNA methylation and fetal growth. To investigate this hypothesis, we supplemented sows with melatonin during mid-gestation and performed reduced representation bisulfite sequencing (RRBS) to characterize placental DNA methylation profiles. We further integrated the methylation and transcriptomic data to identify candidate genes showing coordinated alterations in DNA methylation and gene expression.

## 2. Results

### 2.1. Effects of Melatonin Supplementation on the Incidence of IUGR and Reproductive Performance of Sows

**Figure 1:**
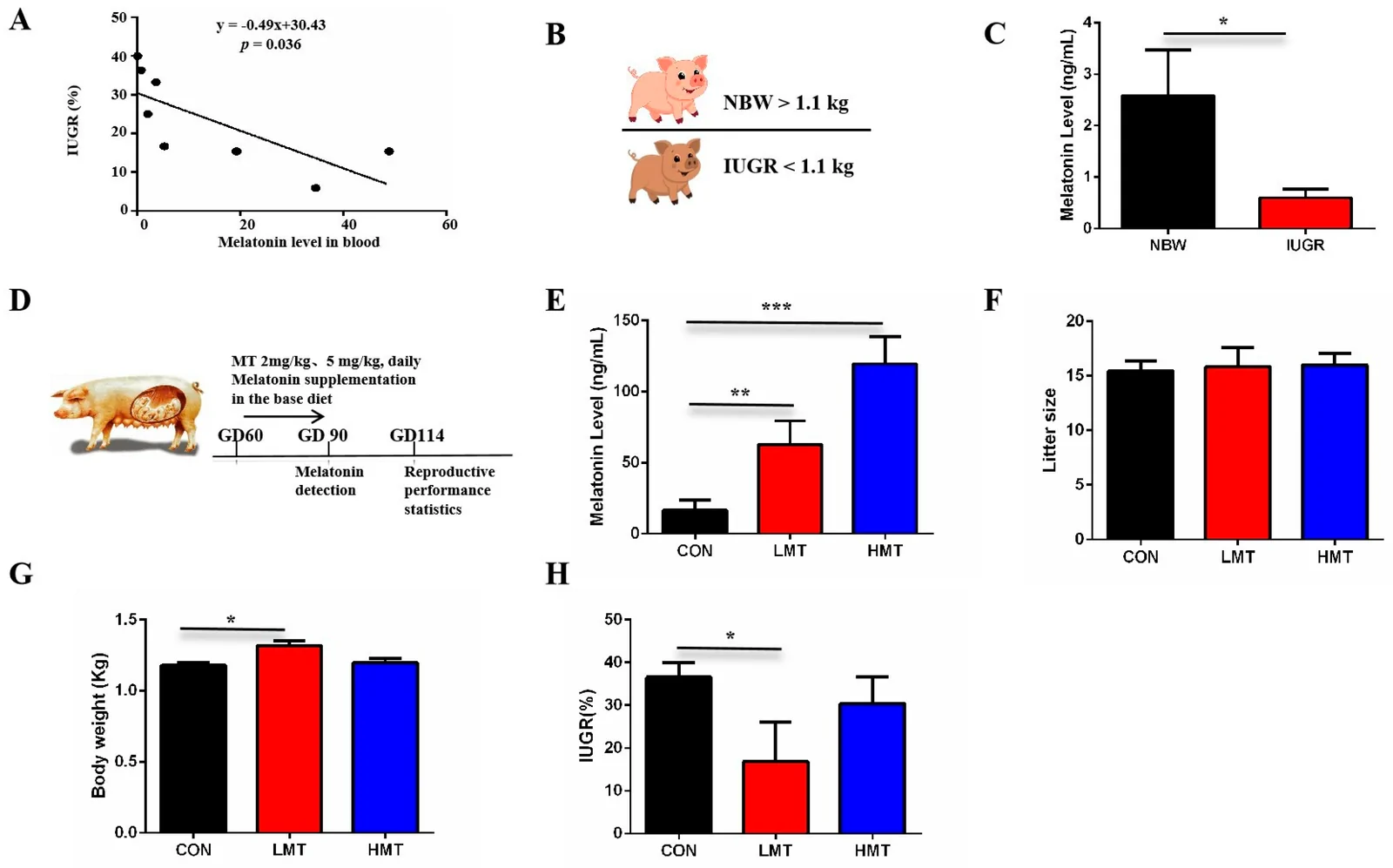
The effects of melatonin supplementation on the IUGR and reproductive performance of pigs. (**A**) Correlation between melatonin concentration in the peripheral blood of pregnant sows and the IUGR rate of piglets. (**B**,**C**) Statistics of melatonin concentration in umbilical cord blood of piglets with different birth weights (n = 7). (**D**) Experimental scheme for exogenous melatonin supplementation during gestation (n = 10). (**E**) Detection of melatonin concentration in the peripheral blood of sows after melatonin supplementation (n = 10). (**F**–**H**) Average litter size of sows after melatonin supplementation (n = 6), average birth weight of piglets (CON, n = 90, LMT, n = 95, HMT, n = 96) and IUGR rate (n = 6). Ten sows were initially included in each experimental group. Three sows per group were randomly sacrificed on gestational day 90 for placental tissue collection, and six sows from the remaining animals were randomly selected for reproductive performance analysis. Thus, n = 10 for the treatment-related analysis and n = 6 sows/litters for the reproductive-performance analysis. CON = control group, LMT = low melatonin (2 mg/kg) group, HMT = high melatonin (5 mg/kg) group. Data are presented as means ± SEM, * *p* < 0.05, ** *p* < 0.01, *** *p* < 0.001.

By measuring the concentration of melatonin in the peripheral blood of pregnant sows and comparing it with the ratio of IUGR piglets per litter after delivery, we observed a significant negative correlation between maternal melatonin levels during pregnancy and the IUGR ratio of piglets (Figure 1A). In addition, the melatonin concentration in the umbilical cord blood of normal-birth-weight piglets was significantly higher than that of IUGR piglets, further supporting this negative correlation between melatonin levels and the occurrence of IUGR in piglets (Figure 1B,C). Based on these results, two doses of melatonin (2 and 5 mg/kg) were selected for administration to sows from days 60 to 90 of gestation to compare the effects of different melatonin doses. The results showed that levels of melatonin in the peripheral blood of sows increased significantly with both doses compared to the control sows (Figure 1D,E), and the treatment did not affect the litter size (Figure 1F). The supplementation of 2 mg/kg of melatonin significantly increased the average birth weight of piglets and reduced the ratio of IUGR piglets compared to the control (Figure 1G,H). The supplementation with a higher dose of melatonin (5 mg/kg) slightly increased the average birth weight or the ratio of IUGR piglets, but these increases failed to reach a significant difference compared to the control (Figure 1G,H).

### 2.2. Effects of Melatonin on Genome-Wide DNA Methylation Profiling and Patterns in Placenta

In order to analyze the mechanism by which melatonin supplementation during pregnancy reduced the IUGR ratio in piglets, we performed reduced representation bisulfite sequencing (RRBS) to characterize the DNA methylation profiles of placentas from four groups: the control group with normal-birth-weight piglets (CN), the control group with intrauterine growth restriction (IUGR) piglets (CI), the melatonin-treated group with normal-birth-weight piglets (MN), and the melatonin-treated group with IUGR piglets (MI). After stringent quality control, including the removal of adapter-contaminated reads, low-quality reads, and reads containing Ns (undetermined bases), 33,272,897 to 50,695,963 clean reads were obtained from each of the 16 DNA libraries. The mapping ratio of clean reads to the pig genome ranged from 76.64% to 81.1% for all sixteen models. The bisulfite conversion rate ranged from 99.535% to 99.602% per sample (Table 1). These results indicate that the sequencing data are of high quality and conducive to bioinformatics analysis.

**Figure 2:**
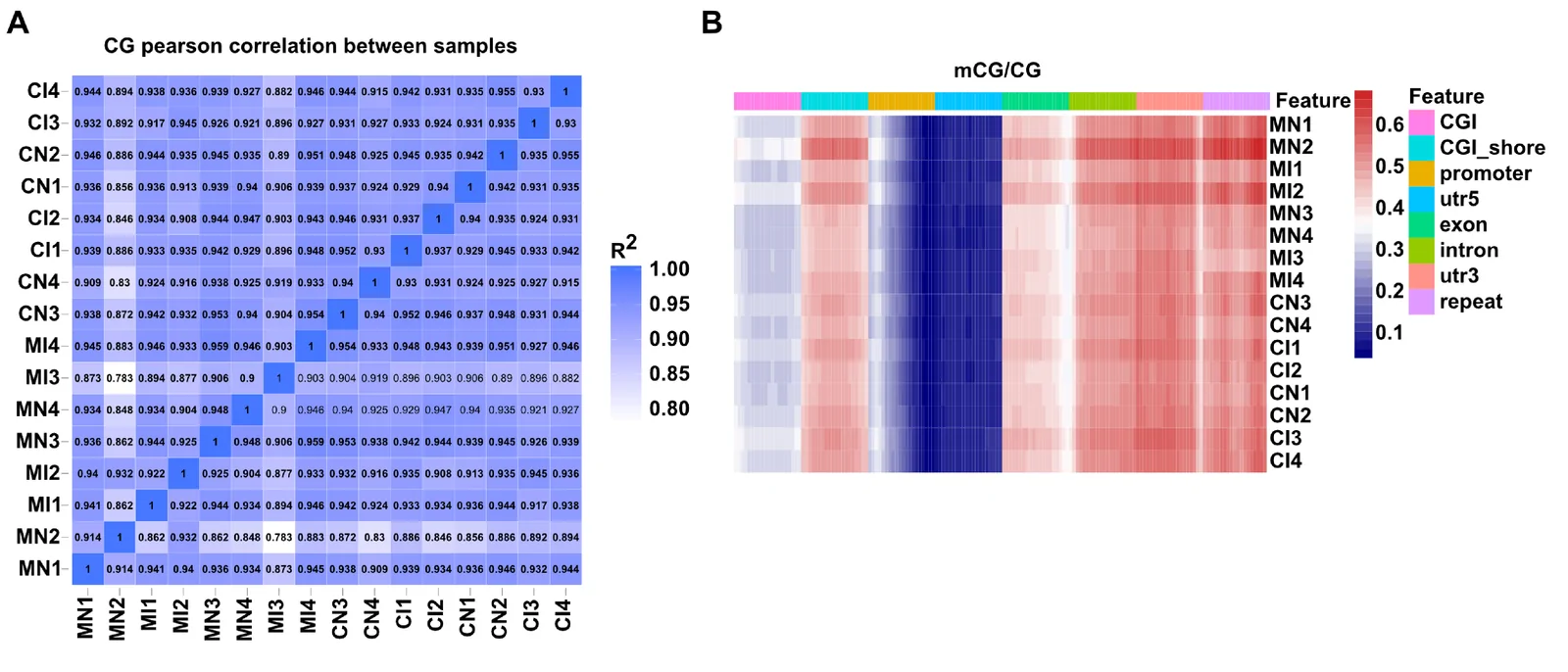
Correlation and clustering heat map analysis. (**A**) Sample correlation analysis. (**B**) Heat map analysis of methylation levels in functional regions between samples. CN = control group with normal-birth-weight piglets, CI = Control group with IUGR piglets, MN = group with melatonin treatment with normal-birth-weight piglets, MI = group with melatonin treatment and IUGR piglets, n = 4 per group.

We statistically analyzed the coverage of C sites, and the average coverage depth of all C sites in the genome of sixteen samples was 17.1375. The distribution patterns of methylated cytosines (mCs) in three sequence contexts—CG (cytosine followed by guanine), CHG, and CHH (where H represents A, C, or T)—revealed that CG methylation was the most prevalent form of methylation in all samples, whereas methylation occurred less frequently in CHG and CHH contexts, so we focused on CpG methylation in the further analysis (Table 2). Pearson correlation analysis of the CpG bases suggested high correlation across all samples (R^2^ > 0.783) (Figure 2A). The results also showed that the methylation level distribution among all samples in the gene function regions was consistent (Figure 2B).

### 2.3. Effects of Melatonin Treatment on Distribution of Methylation Levels in Gene Functional Component Regions

**Figure 3:**
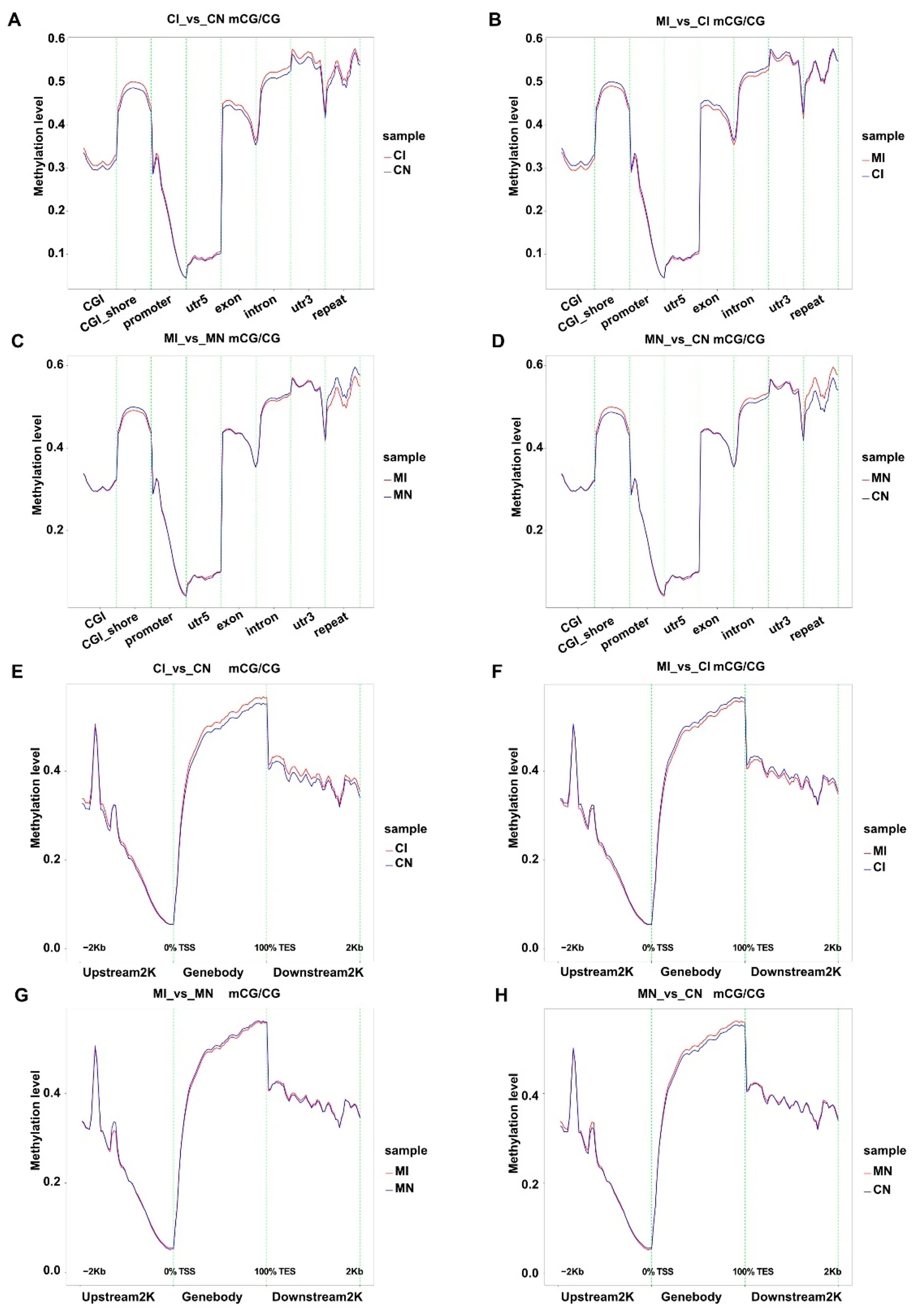
Comparison of methylation-level distribution in different components. (**A**–**D**) Comparison of methylation-level distribution in functional regions between CI vs. CN, MI vs. MN, MI vs. CI and MI vs. MN. (**E**–**H**) Comparison of upstream and downstream methylation levels between CI vs. CN, MI vs. MN, MI vs. CI and MI vs. MN. CN = control group with normal-birth-weight piglets, CI = Control group with IUGR piglets, MN = group with melatonin treatment with normal-birth-weight piglets, MI = group with melatonin treatment and IUGR piglets, n = 4 per group.

We analyzed the distribution of CpG methylation sites in different functional regions of the genome in porcine placental tissues from the CN, CI, MN, and MI groups. As shown in Supplementary Figure S1, CpG methylation in porcine placental tissues of different groups is mainly concentrated in CGI shore, intron, and 3′ untranslated regions. However, from the upstream region to the gene body and downstream areas, the methylation level of mCG decreased and then increased before levelling off (Supplementary Figure S2). These findings reveal the distribution characteristics of CpG methylation in functional gene regions of porcine placental tissues. Additionally, we compared the distribution characteristics of CpG methylation levels in gene functional regions among different groups. Compared to the CN group, the CI group exhibited relatively higher CpG methylation levels in CGI, CGI shore, exon, intron, and 3′ untranslated regions (Figure 3A). In contrast, the MI group showed a distinct CpG methylation pattern compared with the CI group following melatonin supplementation (Figure 3B). Differential methylation between the MI and MN groups, as well as between the MN and CN groups, was mainly distributed in the CGI shore, intron, and repeat regions (Figure 3C,D). Through comparative analysis of the methylation-level distribution characteristics between upstream and downstream regions of genes, it was found that differential methylation between CI and CN, as well as between MI and CI, was mainly concentrated in the gene body and downstream regions (Figure 3E,F). Differential methylation between MI and MN, as well as between MN and CN, was primarily focused in the gene body region (Figure 3G,H).

### 2.4. Effects of Melatonin on the Dynamics of the DNA Methylation Alterations in Swine Placentas

To explore the function and regulatory mechanism of DNA methylation in placenta development, we identified differentially methylated regions (DMRs) between the CN, CI, MN, and MI placentas. There are obvious differences in DMR between different groups, and the length of the DMR region is mainly about 100 bp (Supplementary Figure S3). Compared to the CN group, we identified a total of 1719 DMRs in the CI group, including 1222 hypermethylated DMRs and 497 hypomethylated DMRs. These differential DMRs were annotated to 858 genes, consisting of 651 hypermethylated genes and 271 hypomethylated genes. A total of 1787 DMRs were identified when evaluating the MI and CI piglets’ placenta, including 836 hypermethylated and 951 hypomethylated DMRs. These differential DMRs were annotated to 903 genes, consisting of 453 hypermethylated genes and 522 hypomethylated genes. Next, 2100 DMRs were identified when analyzing the MI vs. MN piglets’ placenta, including 1191 hypermethylated and 909 hypo methylated DMRs. These differential DMRs were annotated to 1061 genes, consisting of 555 hypermethylated genes and 597 hypomethylated genes. All of these DMRs are listed in Table 3.

**Figure 4:**
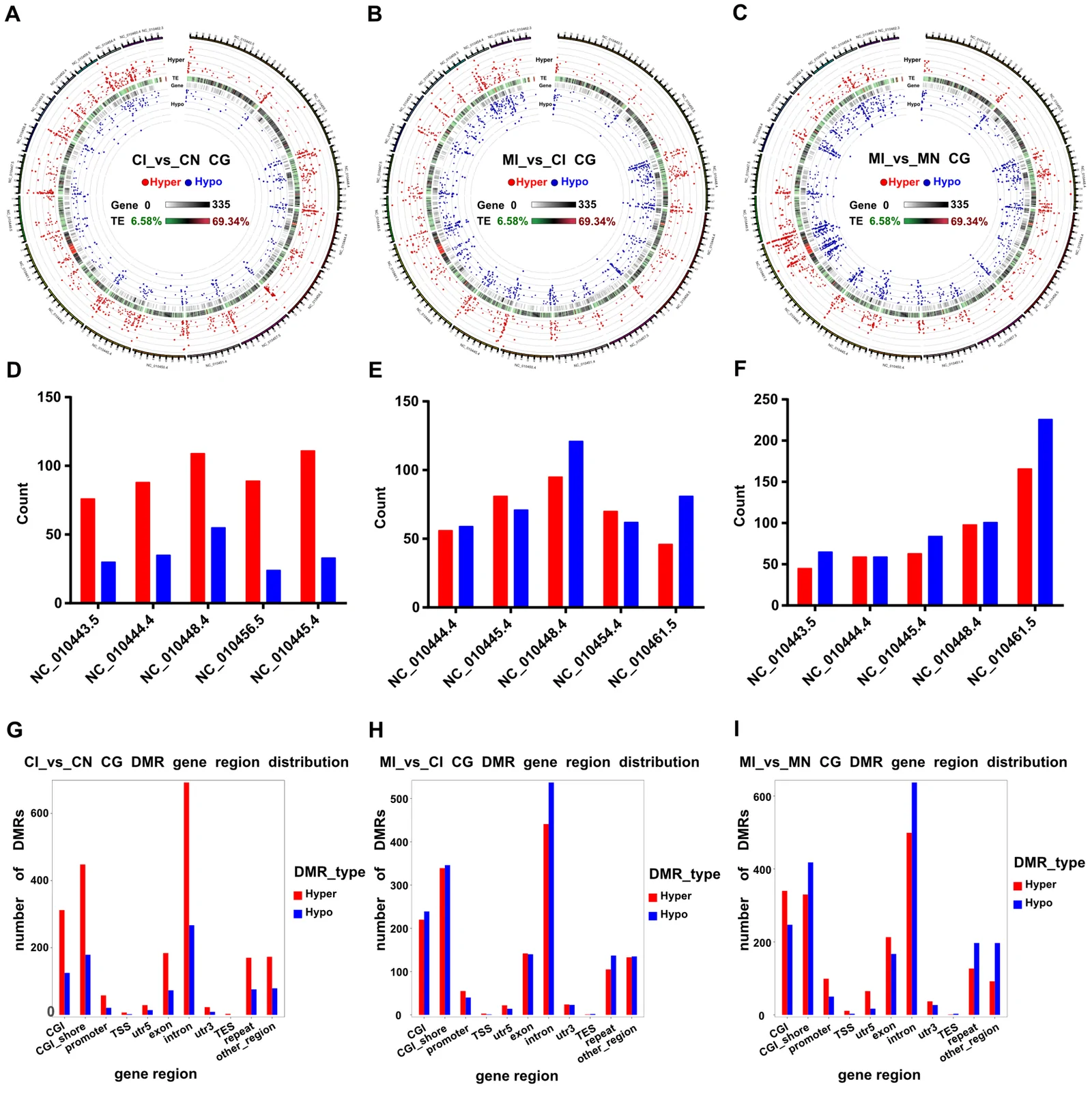
The distribution of differential methylation sites across the genome among groups. (**A**–**C**) The significance and the distribution of DMRs on chromosomes. (**D**–**F**) Chromosomes with the major DMR distribution between different groups. (**G**–**I**,) DMRs anchoring area distribution. CN = control group with normal-birth-weight piglets, CI = Control group with IUGR piglets, MN = group with melatonin treatment with normal-birth-weight piglets, MI = group with melatonin treatment and IUGR piglets, n = 4 per group.

Differential methylation in different groups was represented in a circos plot covering all of the chromosomes. The circos plot represents differentially methylated regions between the groups. The DMRs between the CI and CN groups are primarily located on chromosome NC_010448.4, consisting of 109 hypermethylated DMRs and 55 hypomethylated DMRs (Figure 4A,D). Interestingly, the DMRs between the MI and CI groups are also predominantly found on chromosome NC_010448.4 (chromosomes 6), including 95 hypermethylated DMRs and 121 hypomethylated DMRs (Figure 4B,E). However, the DMRs between the MI and MN groups were primarily located on chromosome NC_010461.5 (chromosomes X), comprising 166 hypermethylated DMRs and 266 hypomethylated DMRs (Figure 4C,F). An analysis of the anchored regional distribution of DMRs among different groups revealed that these DMRs were predominantly found in the intron, CGI, and CGI shore regions of genes, with relatively fewer distributions in the TSS and TES regions (Figure 4G–I).

### 2.5. GO and KEGG Pathway Enrichment Analysis of DMR-Related Genes

**Figure 5:**
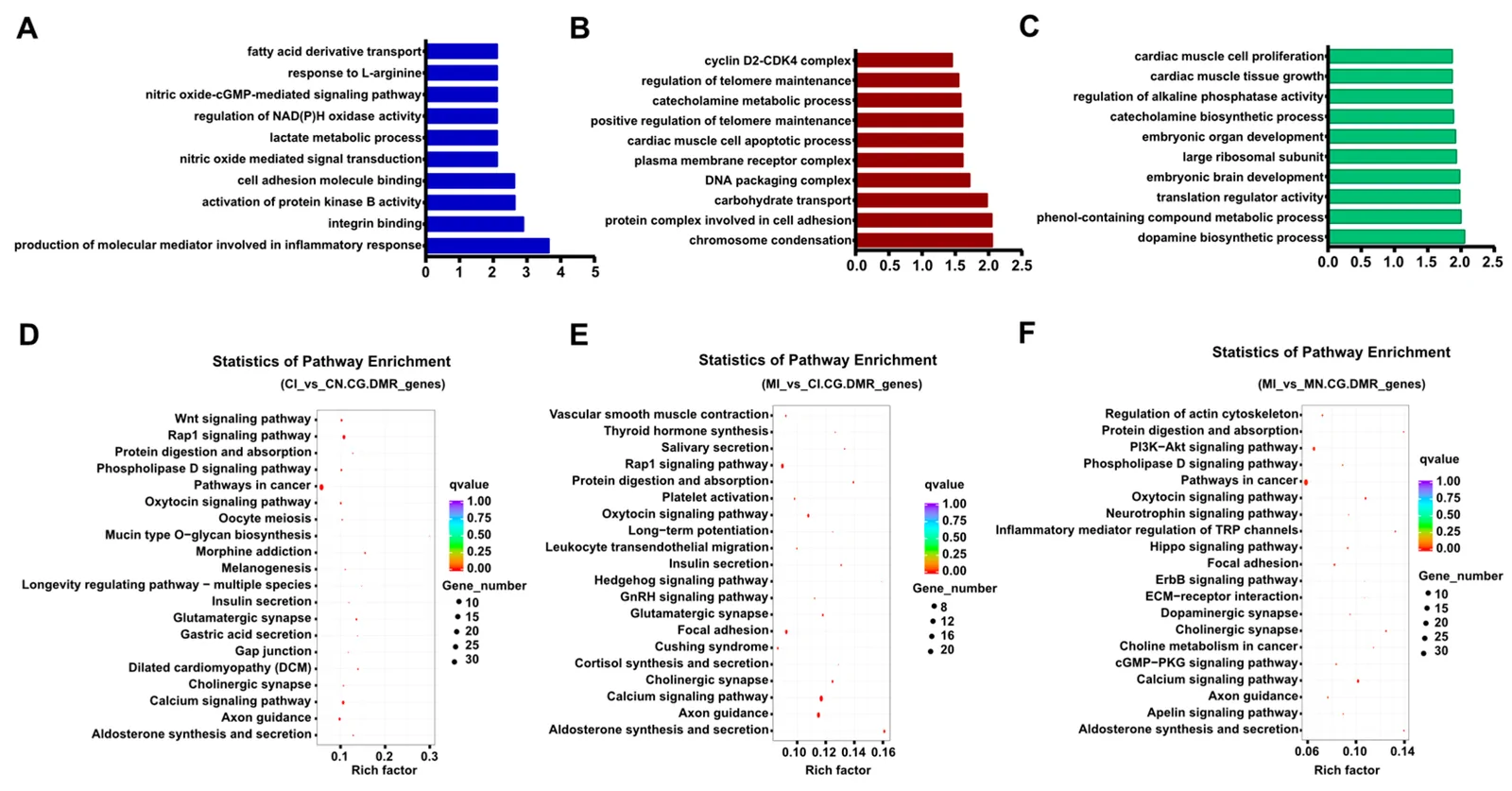
DMR annotation gene enrichment analysis. (**A**–**C**) GO enrichment analysis of DMRs annotated genes between CI vs. CN, MI vs. CI, MI vs. MN groups. (**D**–**F**) KEGG enrichment analysis of DMRs annotated genes between CI vs. CN, MI vs. CI, MI vs. MN groups. CN = control group with normal-birth-weight piglets, CI = Control group with IUGR piglets, MN = group with melatonin treatment with normal-birth-weight piglets, MI = group with melatonin treatment and IUGR piglets, n = 4 per group.

We performed Gene Ontology (GO) and Kyoto Encyclopedia of Genes and Genomes (KEGG) pathway enrichment analyses to determine the potential functions of the DMRs. Our results showed that the DMRs were significantly enriched in several GO terms related to biological processes, cellular components, and molecular functions. The significantly enriched terms between the CI and CN groups include production of mediators involved in inflammatory response, integrin binding, activation of protein kinase B activity, cell adhesion molecule binding, and nitric oxide-mediated signal transduction (Figure 5A). The differential DMRs between the MI and CI groups are primarily enriched in entries, including chromosome condensation, protein complex involved in cell adhesion, carbohydrate transport, positive regulation of telomere maintenance, and lactate metabolic process (Figure 5B). Meanwhile, the differential DMRs between the MI and MN groups are mainly enriched in the dopamine biosynthetic process, translation regulator activity, embryonic organ development, catecholamine biosynthetic process, and transporter activity (Figure 5C).

The KEGG pathway enrichment analysis showed that the significantly enriched pathways between the CI and CN groups include the wnt signaling pathway, Rap1 signaling pathway, and protein digestion and absorption (Figure 5D). The differential DMRs between the MI and CI groups are primarily enriched in pathways such as vascular smooth muscle contraction, thyroid hormone synthesis, and Rap1 signaling pathway (Figure 5E). The differential DMRs between the MI and MN groups are mainly enriched in pathways like regulation of actin cytoskeleton, protein digestion and absorption, and PI3K-Akt signaling pathway (Figure 5F).

### 2.6. Integrated Analysis of DMRs and DEGs

**Figure 6:**
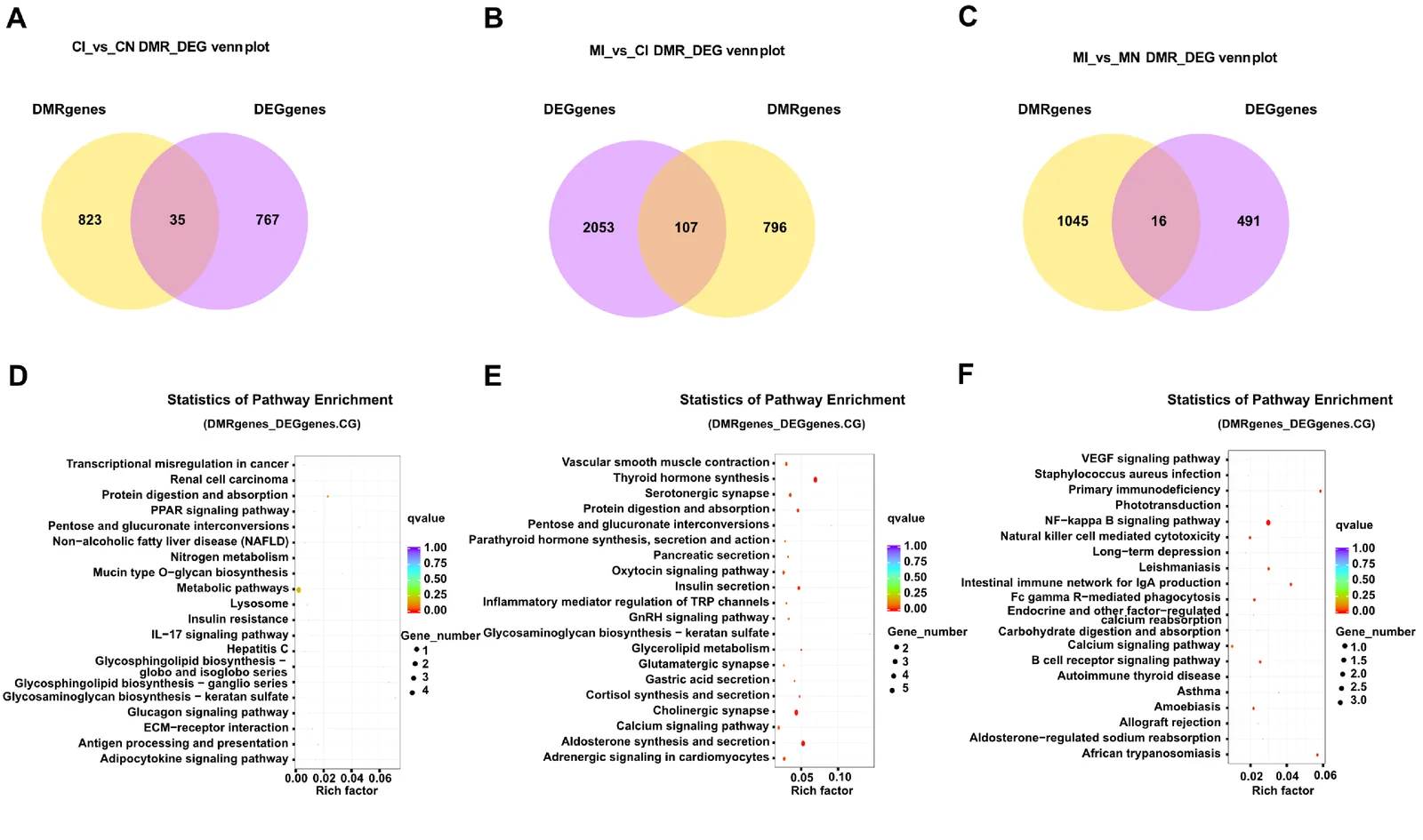
The results of integrated analysis of DMRs and DEGs. (**A**–**C**) Venn diagram of common differential genes of DMRs and DEGs. (**D**–**F**) KEGG enrichment analysis of common differential genes in methylation and transcriptome. CN = control group with normal birth-weight-piglets, CI = Control group with IUGR piglets, MN = group with melatonin treatment with normal-birth-weight piglets, MI = group with melatonin treatment and IUGR piglets.

**Figure 7:**
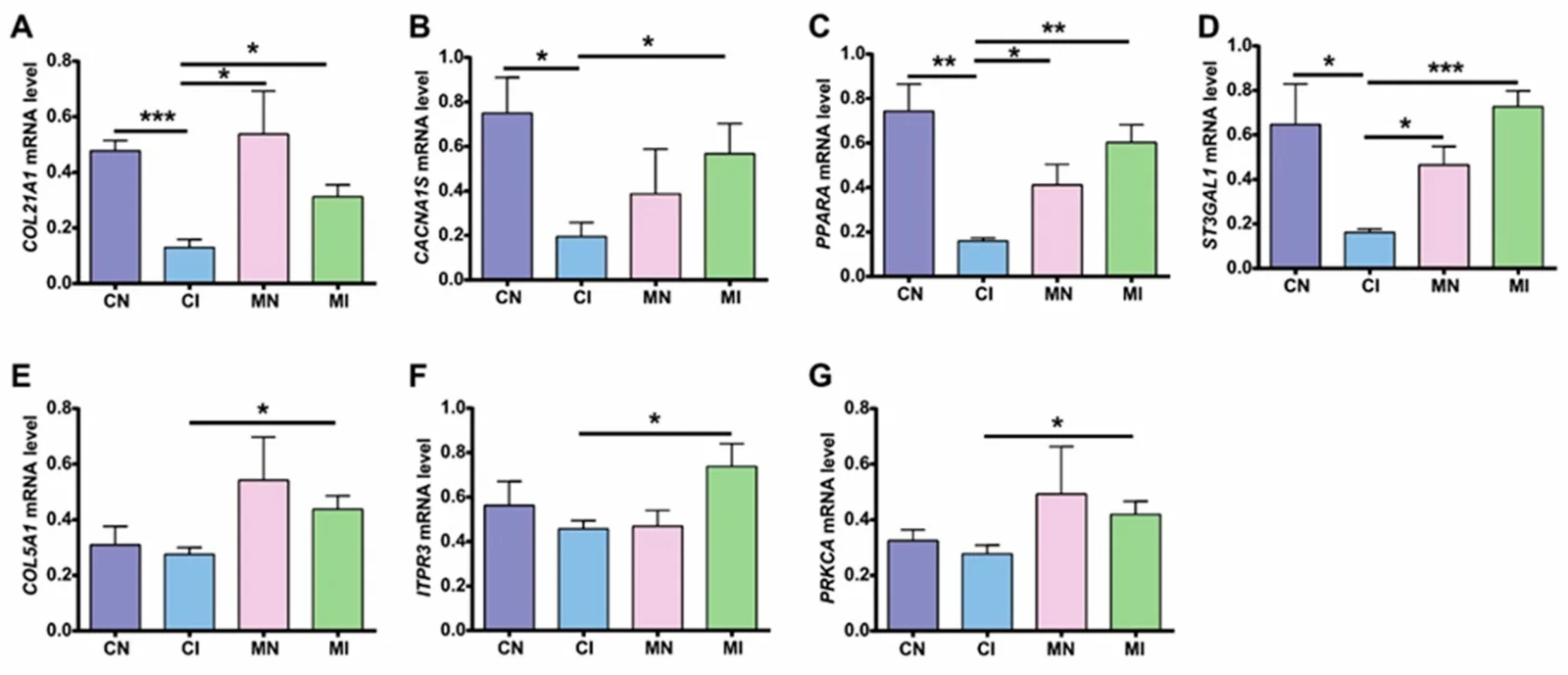
The gene expression level was detected by qPT-PCR. (**A**–**G**) The mRNA levels of COL21A1, CACNA1S, PPARA, ST3GAL3, COL5A1, ITPR3 and PRKCA in placental tissue. CN = control group with normal-birth-weight piglets, CI = Control group with IUGR piglets, MN = group with melatonin treatment with normal-birth-weight piglets, MI = group with melatonin treatment and IUGR piglets, n = 4 per group. Data are presented as means ± SEM, * *p* < 0.05, ** *p* < 0.01, *** *p* < 0.001.

To further investigate the mechanism underlying the effect of melatonin supplementation during pregnancy on alleviating IUGR in fetal pigs, we conducted an integrated analysis of placental methylation and transcriptome sequencing results from four groups of CN, CI, MI, and MN. A Venn diagram analysis of DMRs and differentially expressed genes (DEGs) between the CI and CN groups revealed 35 overlapping genes. KEGG enrichment analysis indicated that these overlapping genes were primarily associated with pathways, including protein digestion and absorption (COL21A1, LOC110259172), PPAR signaling pathway (PPARA) and mucin-type O-Glycan biosynthesis (ST3GAL1) (Figure 6A,D). Between the MI and CI groups, 107 overlapping genes were identified from DMRs and DEGs, which were mainly enriched in pathways, including protein digestion and absorption (COL5A1, COL21A1) and vascular smooth muscle contraction (CACNA1S, ITPR3, and PRKCA) (Figure 6B,E). Between the MI and MN groups, 16 overlapping genes were found, primarily enriched in signaling pathways such as the VEGF signaling pathway (PRKCB) and lysine degradation (TNFRSF13C, PRKCB and LOC102165485) (Figure 6C,F). qRT-PCR results indicated that, compared to the CN group, the mRNA levels of COL21A1, CACNA1S, PPARA, and ST3GAL1 in the placenta were significantly reduced in the CI group, but these gene expression levels could be partially restored after melatonin supplementation (Figure 7A–D) (*p* < 0.05). Additionally, the expression levels of COL5A1, ITPR3, and PRKCA were significantly elevated in the MI group (Figure 7E–G) (*p* < 0.05).

## 3. Discussion

The placenta serves as a “transport station” and plays a crucial role in transporting nutrients, oxygen/carbon dioxide, and metabolic waste between the mother and the fetus. Therefore, the health of the placenta is a prerequisite for the normal growth and development of the fetus [30]. As a result, a placental abnormality will lead to poor fetal development and growth, manifesting as low birth weight and growth delays referred to as IUGR. IUGR is associated with a decreased growth rate, increased fetal morbidity and mortality in fetuses [31,32]. Currently, effective interventions to prevent or reduce the incidence of IUGR remain limited. Understanding the underlying mechanisms of IUGR is, therefore, essential for developing effective preventive and therapeutic strategies. In the current study, we systematically investigated the potential relationship between placental alterations and the incidence of intrauterine growth restriction (IUGR) in pigs. In particular, we examined the association between placental DNA methylation patterns and IUGR, as well as the potential beneficial effects and underlying molecular mechanisms of melatonin supplementation during gestation. We noticed that melatonin exhibits profound effects on DNA methylation and it also has a variety of biological functions, including the regulation of circadian rhythm, antioxidation, and anti-inflammation [33,34,35]. Importantly, the placenta possesses a local melatonergic system, including the melatonin-synthesizing enzymes aralkylamine N-acetyltransferase (AANAT) and acetylserotonin O-methyltransferase (ASMT), as well as the melatonin receptors MTNR1A and MTNR1B, suggesting that melatonin may be locally synthesized and exert its biological effects within placental tissue [36]. Consistent with this notion, previous studies have reported significantly lower cord blood melatonin concentrations in pregnancies complicated by IUGR than in normal pregnancies [24]. Furthermore, a positive correlation between placental melatonin concentrations and fetal birth weight was observed [23]. We also observed a negative correlation between peripheral blood melatonin concentrations in sows and the incidence of IUGR. Consistent with previous findings, IUGR piglets exhibited significantly lower cord blood melatonin concentrations than normally grown piglets. It is well documented that stressful conditions such as high temperatures, toxic substances, and hypoxia can cause IUGR in fetuses, while melatonin administration alleviates fetal growth impairment by improving gut microbiota distribution, inhibiting oxidative stress, and modulating cellular autophagy [22,37,38]. As a polytocous animal, pigs have a naturally high rate of IUGR, which can reach up to 25%, making them a natural model for studying IUGR [39]. Peng and colleagues (2021) reported that melatonin supplementation from early pregnancy to parturition in sows reduced the proportion of piglets with a birth weight lower than 900 g [40]. This beneficial effect was suggested to be associated with the antioxidant properties of melatonin. However, it should be noted that reactive oxygen species (ROS) play an important physiological role in placental vascular development during early gestation, suggesting that excessive antioxidant supplementation may not always be beneficial. Indeed, previous studies using pregnancy-related disease models, such as preeclampsia, have suggested that melatonin supplementation during early gestation may aggravate fetal growth restriction under certain conditions [41]. In our previous study, we demonstrated that supplementation with 2 mg/kg melatonin during late gestation in sows, a period characterized by rapid fetal growth, improved placental antioxidant capacity, promoted fetal growth, and increased piglet birth and weaning weights [25]. However, there are no reports on whether melatonin supplementation during the middle gestation stage of sows will impact fetal development. In this study, we found that melatonin supplementation from day 60 to day 90 of pig gestation at a dose of 2 mg/kg significantly increased the average birth weight and reduced the rate of IUGR in piglets. Interestingly, increasing the melatonin dose from 2 to 5 mg/kg did not result in a further improvement in reproductive outcomes. Although both doses significantly increased circulating melatonin concentrations in the sows, only the 2 mg/kg treatment significantly increased the average birth weight of piglets and reduced the proportion of IUGR piglets. These findings suggest that the beneficial effects of melatonin during mid-gestation may not follow a simple linear dose–response relationship. Melatonin exerts multiple physiological actions through receptor-dependent signaling as well as receptor-independent antioxidant mechanisms, and excessive supplementation may not necessarily provide additional benefits once an effective physiological range has been reached. Moreover, an appropriate balance of oxidative and redox signaling is required for normal placental development and angiogenesis; therefore, increasing the dose of an antioxidant such as melatonin may not proportionally enhance placental function. Nevertheless, because only two melatonin doses were examined in the present study, the precise mechanism underlying the different responses to 2 and 5 mg/kg melatonin remains unclear. Further dose–response studies are required to determine the optimal dose and therapeutic window of melatonin supplementation during mid-gestation.

DNA methylation is a common epigenetic modification in mammals and plays an important role in reproductive processes, including oocyte meiotic maturation and embryonic development. Generally, DNA methylation can suppress gene expression by altering chromatin accessibility and influencing transcription factor binding. In porcine placentas, Tan et al. [14] reported significant changes in the methylation patterns of genes associated with placental development at different gestational stages, suggesting their potential involvement in fetal development. Abnormal placental DNA methylation patterns have also been associated with alterations in placental angiogenesis, extracellular matrix remodeling, and low birth weight in piglets [25]. Consistent with these findings, we observed higher levels of placental DNA methylation in IUGR piglets than in normally grown piglets, suggesting an association between altered placental DNA methylation patterns and impaired fetal growth. Melatonin has profound effects on epigenetic regulation, particularly on DNA methylation [42], and stimulates cell differentiation [43]. In contrast to increased DNA methylation in the placenta of an IUCR fetus, melatonin significantly reduces methylation levels (5-mC) by downregulating the expression of DNA methyltransferase 1 (DNMT1) and methyl-CpG-binding protein 2 (MeCP2) [43]. In the present study, melatonin supplementation during mid-gestation was associated with alterations in placental DNA methylation patterns across multiple genomic regions, including CpG islands (CGIs), CGI shores, exons, introns, and 3′ untranslated regions (3′ UTRs). These changes in DNA methylation were observed alongside improvements in fetal growth outcomes, suggesting a possible relationship among melatonin supplementation, placental DNA methylation, and fetal development. However, these findings are primarily associative and do not establish whether the observed DNA methylation changes directly contribute to the reduced incidence of IUGR.

With the integrated analysis of DMRs and DEGs, a total of 35 differentially expressed genes were identified in the placentas between normal-birth-weight and IUGR piglets. These genes were enriched in pathways such as protein digestion and absorption, nitrogen metabolism, PPAR signaling pathway, and mucin-type O-Glycan biosynthesis. Within the protein digestion and absorption pathway, the COL21A1 gene was specifically enriched. The placentas of IUGR piglets exhibited higher methylation levels of COL21A1, accompanied by lower COL21A1 mRNA expression. In contrast, melatonin supplementation was associated with lower COL21A1 methylation levels and higher COL21A1 mRNA expression. These coordinated changes suggest a potential relationship between COL21A1 methylation and expression, although a direct causal relationship remains to be established. COL21A1 is a crucial component of the extracellular matrix (ECM), involved in regulating the assembly and stability of the ECM. The interaction between cells and the ECM is essential for placental development [44]. For example, previous studies have reported significantly lower placental COL1A2 expression in IUGR piglets than in normal-birth-weight piglets [25]. Insufficient placental blood supply is an important factor contributing to placental dysfunction and IUGR and may be associated with disturbances in nitric oxide (NO) metabolism. NO is a signaling molecule involved in regulating placental vascular tone and uteroplacental blood flow [45]. Both animal and human studies have suggested that decreased nitric oxide (NO) levels are associated with IUGR [46]. In the present study, melatonin supplementation was associated with altered methylation and expression of several genes, including CACNA1S, ITPR3, and PRKCA, which are enriched in the vascular smooth muscle contraction pathway. Given the established importance of vascular regulation and NO signaling in placental function, these coordinated molecular alterations may be relevant to placental vascular function. However, NO production and placental blood flow were not directly measured in the present study, and the functional consequences of these molecular changes, therefore, require further investigation. In addition, peroxisome proliferator-activated receptor alpha (PPARA) is involved in the regulation of lipid metabolism, fatty acid oxidation, oxidative stress, and pathways associated with NO production and angiogenesis [47]. Previous animal studies have shown that maternal obesity is associated with reduced placental PPARA expression and altered fatty acid oxidation. Maternal adiponectin supplementation has been reported to increase placental PPARA expression and alleviate obesity-associated placental dysfunction, suggesting a potential role for PPARA in maintaining placental function and supporting fetal development [48]. In this study, we found that PPARA, a gene involved in the PPAR signaling pathway, exhibited increased DNA methylation and decreased mRNA expression in the placentas of IUGR piglets compared with those of normal-birth-weight piglets. These findings suggest that altered PPARA methylation and expression may be associated with impaired placental metabolic regulation in IUGR. However, the functional contribution of PPARA to placental dysfunction and fetal growth restriction requires further investigation. Furthermore, the abnormal increase in the methylation level of the ST3 beta-galactoside alpha-2,3-sialyltransferase 1 (ST3GAL1) gene, enriched in the mucin-type O-Glycan biosynthesis pathway, leads to a decrease in its expression level in the IUGR condition. ST3GAL1 is a sialyltransferase that catalyzes the transfer of sialic acid from CMP-sialic acid to the Galβ1-3GalNAc substrate, involving processes such as embryonic development, neural development, and immune responses [49,50]. Previous studies have reported that disruption of certain sialylation pathways in mice can result in embryonic lethality around embryonic day 9.5 (E9.5), highlighting the importance of sialylation in embryonic development [51,52]. These observations suggest that altered ST3GAL1 expression may be relevant to placental function and fetal growth. However, its specific role in porcine placental development and IUGR remains to be elucidated. It should be noted that placental melatonin concentrations and the expression of melatonin receptors MTNR1A and MTNR1B were not examined in the present study. Therefore, whether the observed alterations in placental DNA methylation and gene expression represent direct effects of melatonin signaling within the placenta or indirect systemic effects remains unclear. Further studies examining placental melatonin signaling will be required to distinguish these possibilities.

## 4. Materials and Methods

### 4.1. Animal Ethics

All the experimental procedures applied in this study were reviewed and approved by the Animal Welfare Committee of China Agricultural University (Permit Number: AW60103202-3-1). All procedures involving handling, management, and health care of live birds followed the regulations of laboratory animals used for scientific purposes and were implemented within the China Agricultural University. We also confirmed that all experiments were compliant with the Animal Research Reporting of In Vivo Experiments (ARRIVE) guidelines.

### 4.2. Animals and Experimental Design

The sows (French hybrid) were sourced from Beijing Fengsenyuan Agricultural Development Co., Ltd. (Beijing, China). All sows were primiparous (approximately 1-year-old) with similar backfat thickness. Thirty pregnant sows with similar body weights (around 200 kg) at 60 days of gestation were selected and divided into a control group, a low-dose melatonin group (LMT, 2 mg/kg), and a high-dose melatonin group (HMT, 5 mg/kg), with 10 sows initially included in each group. The dose of 2 mg/kg was selected based on our previous study, demonstrating beneficial effects of this dose during late gestation [25], whereas 5 mg/kg was included as a higher dose to evaluate whether increased melatonin supplementation could provide additional benefits. The environmental light/dark cycle was set to 12/12 h, and the temperature was controlled between 20 and 25 °C. These sows were given the same basal diet (feed ingredients: crude protein ≥ 15%, crude fiber ≤ 8%, crude ash content ≤ 9%, the total phosphorus ≥ 0.6%, L—lysine ≥ 0.6%, water ≤ 14%, calcium 0.8–1.3%, sodium chloride 0.3–0.8%) at 8 a.m. and 5 p.m. daily (each feeding is approximately 3 kg), but the treated group received melatonin. Melatonin was administered at 8 a.m. every morning at two doses of 2 mg/kg and 5 mg/kg, directly added to their diet to ensure complete consumption [25]. The treatment lasted from the 60th day to the 90th day of gestation. On the 90th day, three sows were randomly selected and sacrificed in each group to collect placentas. On the 107th day of gestation, the rest of the sows were transferred to the farrowing house to prepare their delivery. Among the remaining sows, six sows from each group were randomly selected for the analysis of reproductive performance. During farrowing, we recorded the litter size, the birth weight of piglets, and the IUGR rate for each group of sows (6 sows per group). Piglets with a birth weight <1.1 kg were classified as IUGR piglets, whereas piglets with a birth weight ≥1.1 kg were classified as normal-birth-weight (NBW) piglets.

### 4.3. Blood Collection

To avoid excessive stress on the sows caused by frequent blood sampling, we decided to minimize the number of blood collections. Therefore, blood was collected before and after melatonin treatment (corresponding to the mid to late phase of the melatonin treatment cycle), specifically on the 60th and 90 days of gestation, respectively. For each collection, 8 mL of antecubital venous blood was drawn and placed in a 10 mL sterile heparinized vacuum tube. The blood samples were immediately centrifuged at 3500 g for 15 min to separate the plasma, which was then stored at –80 °C for further analysis of melatonin.

### 4.4. Postnatal Weight Measurement and Placental Tissue Collection of Piglets

At 90 days of gestation, three sows from each group were slaughtered to count and weigh the total number of fetuses, and placental tissues were collected for further analysis. For the remaining sows, the total number of piglets was recorded after birth, and the weights of the piglets were measured after their coats dried. The expelled placental tissues were also collected. The placental tissues were rinsed with physiological saline and immediately placed in liquid nitrogen for rapid freezing (approximately 5 g per placenta, taken from 3 to 4 cm away from the umbilical cord insertion point).

### 4.5. Melatonin Detection

The serum was mixed with methanol at a ratio of 1:4, vortexed, and centrifuged 12,879 g at 4 °C, for 10 min. The supernatant was collected and then filtered through a 0.22 µm filter for the detection of melatonin. Melatonin detection was carried out at the Central Laboratory of the Beijing Institute of Animal Sciences, Chinese Academy of Agricultural Sciences, using high-performance liquid chromatography–mass spectrometry (Agilent 1290-G6470, Santa Clara, CA, USA).

### 4.6. Transcriptome Sequencing of Placental Tissue

Total RNA was extracted from placental tissue using TRIzol (Ambion, San Francisco, CA, USA, 15596026), and genomic DNA was removed using DNase I (TaKara, Shiga, Japan). The quality of RNA samples was assayed using 2100 Bioanalyser (Agilent, Palo Alto, CA, USA) and ND-2000 (NanoDrop Technologies, Wilmington, DE, USA). Once the samples met the qualification criteria (OD260/280 = 1.8–2.2, OD260/230 ≥ 2.0, RIN ≥ 6.5, 28S:18S ≥ 1.0, >2 mg), they were sequenced. The TruSeqTM RNA Sample Preparation Kit (Illumina, San Diego, CA, USA) was used to construct the RNA library. The SuperScript Double-Stranded cDNA Synthesis Kit (Invitrogen, Carlsbad, CA, USA) was employed for reverse transcription to synthesize cDNA and form a stable double-stranded structure. After PCR enrichment of the cDNA using the Sample Preparation Kit (Illumina, San Diego, CA, USA), DNA clean Beads were used to screen for 200–300 bp bands. Following quantification with TBS380 (Picogreen, Eugene, OR, USA), high-throughput sequencing was performed using the Illumina HiSeq XTEN/NovaSeq 6000 (Illumina, Inc., San Diego, CA, USA) sequencing platform, with a read length of PE150.

### 4.7. Methylation Analysis and Reduced Representation Bisulfite Sequencing (RRBS) Library Construction and Sequencing

RRBS analysis was performed on selected placental samples from intrauterine growth restriction (IUGR) and normal placenta samples (4 samples were randomly selected from each group). Firstly, the detection of DNA samples involves agarose gel electrophoresis to analyze the degree of DNA degradation and the presence of RNA contamination. Nanodrop is used to assess the purity of DNA (OD260/280 ratio), while Qubit provides precise quantification of DNA concentration. After the samples passed quality control, the library was constructed using the Illumina Pair-End protocol. Approximately 1 μg of DNA was mixed with unmethylated lambda DNA and digested with MspI enzyme at 37 °C for 16 h. The CpG coverage was 80%, and the duplication rate was 10%. A certain proportion of negative control (lambda DNA) was added. Initially, the DNA samples were digested using the methylation-insensitive restriction enzyme MspI. The digested DNA fragments were then subjected to end repair, A tailing, and ligation with sequencing adapters that had all cytosines methylated. Gel cutting was performed to select DNA fragments with an insert size ranging from 40 to 220 bp (Meisper 2008). Following this, DNA was purified using the MinElute PCR Purification Kit (Qiagen, Hilden, Germany). Subsequently, bisulfite treatment was performed using the EZ DNA Methylation Gold Kit (ZymoResearch, Irvine, CA, USA). Following the treatment, unmethylated cytosines (C) were converted to uracil (U), which became thymine (T) after PCR amplification, while methylated cytosines remained unchanged. PCR was then carried out in a final reaction volume of 50 μL, consisting of 20 μL of adapter-ligated DNA, 4 μL of 2.5 mmol/L dNTPs, 5 μL of 10× buffer, 0.5 μL of JumpStart™ Taq DNA polymerase (Sigma-Aldrich, St. Louis, MO, USA), 2 μL of PCR primers, and 18.5 μL of water. The thermal cycling program was as follows: initial denaturation at 94 °C for 1 min, followed by 5 cycles of denaturation at 94 °C for 10 s, annealing at 62 °C for 30 s, and extension at 72 °C for 30 s. This was repeated for a total of 12 cycles, with a final extension step at 72 °C for 5 min. Afterward, the PCR products were maintained at 12 °C to obtain the final DNA library. After the library construction was completed, initial quantification was performed using Qbit 2.0 (Life Technologies, Carlsbad, CA, USA). The library was then diluted to 1 ng/mL, followed by the assessment of insert fragment length using the 5400 Fragment Analyzer System. Once the results meet expectations, the effective concentration of the library is accurately quantified using the Q-PCR method (with a target effective concentration of >2 nM) to ensure library quality. After passing the library quality control, different libraries were pooled according to the effective concentration and the required target data output for sequencing on the Illumina HiSeq/MiSeq platform (Illumina, Inc., San Diego, CA, USA). The fundamental principle of sequencing is Sequencing by Synthesis. In the sequencing flow cell, four types of fluorescently labeled dNTPs, DNA polymerase, and adapter primers were added for amplification. During the extension of the complementary strand in each sequencing cluster, the incorporation of each fluorescently labeled dNTP releases a corresponding fluorescence signal. The sequencer captures these fluorescence signals, which are then converted into sequencing peaks by computer software, thereby obtaining the sequence information of the fragments under investigation.

### 4.8. Raw Data Filtration and Data Processing

The primary objective of data filtering is to eliminate low-quality data and ensure the quality of clean data. The trimming method was used to remove sequencing adapters and low-quality segments from the sequencing data, enabling more efficient utilization of sequencing data for subsequent analyses based on clean data. The initial data trimming process was performed by the fastq software to trim adapter contaminations and reads with either a head or tail quality below 3 or those containing ‘N’ (where ‘N’ denotes uncertain base information). Thereafter, a sliding window method was used to further trim low-quality reads, discarding those shorter than 36 nucleotides (nt) or those unable to form pairs. Finally, the first 10 base pairs were removed to establish both the single-cell methylation and low-input methylation libraries. Then, the Bismank (Kmeger, 2011, invoking Bowtie2 at its core) was used for the alignment analysis of methylated data against the reference genome. Both the genome sequence and sequencing reads underwent C-to-T and G-to-A conversions, respectively. The converted reads were then aligned against the similarly converted genomic DNA. The best alignment result was selected from among four parallel alignment outcomes as the final results. Differentially methylated regions (DMRs) were identified using DSS software. CpG sites with a minimum sequencing coverage of 10× were included in the analysis. DMRs were defined according to a minimum methylation difference of 20% and an FDR < 0.05.

### 4.9. Quantitative Real-Time PCR (qRT-PCR)

Total RNA was extracted from fresh porcine placenta tissues using TRIzol reagent (Invitrogen, USA). After quality testing, 500 ng of total RNA was synthesized using PrimeScript™RT Master Mix (TaKaRa, Japan) kit. SYBR Green PCR Master Mix (TaKaRa, Japan) was used for PCR amplification. GAPDH is regarded as an internal reference gene. Subtract the CT value of the internal reference gene from the CT value of the target gene to calculate ΔCT. Subsequently, the relative mRNA expression level of the target gene was calculated using the 2^−(ΔΔCT)^ method. The primers used in this study are shown in Table S1.

### 4.10. Statistical Analysis

Data are expressed as mean ± SEM. Statistical analysis was performed with ANOVA followed by a two-tailed Student’s t-test using GraphPad Prism software version 7.00. A *p*-value < 0.05 was considered statistically significant.

## 5. Conclusions

In this study, we identified distinct placental DNA methylation patterns associated with IUGR in piglets and observed a negative correlation between circulating or umbilical cord blood melatonin concentrations and IUGR incidence. Melatonin supplementation during mid-gestation was associated with increased piglet birth weight, a reduced incidence of IUGR, and alterations in placental DNA methylation and gene expression. Integrated RRBS and transcriptomic analyses identified several candidate genes, including COL21A1, PPARA, CACNA1S, ITPR3, and PRKCA, showing coordinated changes in DNA methylation and mRNA expression. These findings reveal an association between melatonin supplementation, placental epigenetic characteristics, and fetal growth and provide candidate molecular targets for further investigation. However, targeted methylation validation and functional studies are required to determine whether these epigenetic alterations directly contribute to the beneficial effects associated with melatonin supplementation. The results provide additional information to reduce the high incidence of IUGR in pigs and maybe also in other species.

## Figures and Tables

**Table 1:** Summary of RRBS methylation sequencing data.

Group	Clean Reads	Mapped Reads	Mapping Rate (%)	BS Conversion Rate (%)	Q30 (%)
CN-1	34,111,300	26,143,849	76.64	99.553	95.68
CN-2	38,607,045	30,173,563	78.16	99.54	95.42
CN-3	41,337,511	31,764,646	76.84	99.552	95.13
CN-4	35,492,490	27,606,846	77.78	99.587	95.5
CI-1	33,272,897	25,602,327	76.95	99.558	95.62
CI-2	34,334,130	27,127,263	79.01	99.558	95.29
CI-3	36,138,579	28,721,321	79.48	99.539	95.84
CI-4	35,393,391	27,388,173	77.38	99.571	95.47
MN-1	35,161,471	27,734,055	78.88	99.558	95.73
MN-2	50,288,411	39,363,271	78.28	99.568	96.12
MN-3	46,092,544	36,297,187	78.75	99.535	95.54
MN-4	50,695,963	40,314,396	79.52	99.602	94.57
MI-1	43,183,728	34,105,163	78.98	99.567	95.68
MI-2	35,444,291	27,541,913	77.7	99.565	95.65
MI-3	49,301,473	39,448,049	80.01	99.605	94.92
MI-4	34,743,526	28,175,907	81.1	99.545	95.43

Clean reads: The number of reads after QC; Mapped reads: The number of sequencing reads that were successfully aligned to the reference genome; Mapping rate (%): The ratio of the number of reads compared to the total reads; BS conversion rate (%): The rate at which Bisulfite converts C into T; Q30 (%): The percentage of all bases with a Q value greater than 30. CN = control group with normal-birth-weight piglets, CI = control group with IUGR piglets, MN = group with melatonin treatment with normal-birth-weight piglets, MI = group with melatonin treatment and IUGR piglets, n = 4 per group.

**Table 2:** Statistics of methylation status at C site among different groups.

Group	C_covgMean	MeanC (%)	MeanCG (%)	MeanCHG (%)	MeanCHH (%)
CN-1	14.5	8.06	41.47	0.73	0.61
CN-2	16.8	8.43	42.9	0.77	0.63
CN-3	17.7	8.43	42.9	0.75	0.63
CN-4	14.5	8.38	40.63	0.7	0.57
CI-1	14.6	8.49	43.7	0.75	0.64
CI-2	14.8	8.13	41.18	0.72	0.6
CI-3	16.8	8.56	44.47	0.74	0.62
CI-4	15.1	8.59	43.82	0.74	0.61
MN-1	15.2	8.29	41.32	0.73	0.62
MN-2	21.8	9.00	46.11	0.74	0.6
MN-3	20.2	7.83	39.68	0.74	0.62
MN-4	21.6	8.41	41.81	0.69	0.58
MI-1	18.7	8.69	43.92	0.73	0.61
MI-2	14.9	9.85	49.58	0.8	0.67
MI-3	21.4	8.09	40.22	0.67	0.56
MI-4	15.6	7.87	40.02	0.73	0.62

C_covgMean: The average coverage depth of all C sites of the genome; meanC (%): Average methylation levels at all C sites of the genome; MeanCG (%): Average methylation levels in the CG region; MeanCHG (%): Average methylation levels in the CHG region; MeanCHH (%): Average methylation levels in the CHH region. CN = control group with normal-birth-weight piglets, CI = Control group with IUGR piglets, MN = group with melatonin treatment with normal-birth-weight piglets, MI = group with melatonin treatment and IUGR piglets, n = 4 per group.

**Table 3:** Statistics of DMRs and gene annotation results.

Group	Total DMRs	Total Annotated Gene	Hyper_ DMRs	Hyper_Annotated Gene	Hypo_ DMRs	Hypo_Annotated Gene
CI vs. CN	1719	858	1222	651	497	271
MI vs. CI	1787	903	836	453	951	522
MI vs. MN	2100	1061	1191	555	909	597

Total DMRs: The total number of differential methylation regions identified between different groups; Total annotated gene: The number of genes annotated by differential methylation regions was identified between different groups; Hyper/Hypo_DMRs: Hypermethylated/hypomethylated differential methylation regions; Hyper/Hypo_annotated gene: Genes annotated by hypermethylated/hypomethylated differential methylation regions. CN = control group with normal-birth-weight piglets, CI = control group with IUGR piglets, MN = group with melatonin treatment with normal-birth-weight piglets, MI = group with melatonin treatment and IUGR piglets, n = 4 per group.

## Data Availability

The original contributions presented in this study are included in the article/Supplementary Material. Further inquiries can be directed to the corresponding author.

## Supplementary Materials

The following supporting information can be downloaded at: https://www.mdpi.com/article/10.3390/ijms27209046/s1.

## References

[1] Salam R.A., Das J.K., Bhutta Z.A. (2014). Impact of Intrauterine Growth Restriction on Long-Term Health. Curr. Opin. Clin. Nutr..

[2] Wu G., Bazer F.W., Wallace J.M., Spencer T.E. (2006). Board-Invited Review: Intrauterine Growth Retardation: Implications for the Animal Sciences. J. Anim. Sci..

[3] Li Y., Zhang H., Su W., Ying Z., Chen Y., Zhang L., Lu Z., Wang T. (2018). Effects of Dietary Bacillus Amyloliquefaciens Supplementation on Growth Performance, Intestinal Morphology, Inflammatory Response, and Microbiota o Intra-Uterine Growth Retarded Weanling Piglets. J. Anim. Sci. Biotechnol..

[4] Lynegaard J.C., Hales J., Nielsen M.N., Hansen C.F., Amdi C. (2020). The Stomach Capacity is Reduced in Intrauterine Growth Restricted Piglets Compared to Normal Piglets. Animals.

[5] Rehfeldt C., Kuhn G. (2006). Consequences of Birth Weight for Postnatal Growth Performance and Carcass Quality in Pigs as Related to Myogenesis. J. Anim. Sci..

[6] Andrés S., Valdés C., Santos A., Mateo J., Giráldez F.J. (2020). Effects of Birth Weight on Animal Performance, Fattening Traits and Meat Quality of Lambs. Animals.

[7] Malassiné A., Frendo J.L., Evain-Brion D. (2003). A Comparison of Placental Development and Endocrine Functions Between the Human and Mouse Model. Hum. Reprod. Update.

[8] Che L., Hu L., Zhou Q., Peng X., Liu Y., Luo Y., Fang Z., Lin Y., Xu S., Feng B. (2020). Microbial Insight into Dietary Protein Source Affects Intestinal Function of Pigs with Intrauterine Growth Retardation. Eur. J. Nutr..

[9] Stremming J., Chang E.I., Knaub L.A., Armstrong M.L., Baker P.N., Wesolowski S.R., Reisdorph N., Reusch J., Brown L.D. (2022). Lower Citrate Synthase Activity, Mitochondrial Complex Expression, and Fewer Oxidative Myofibers Characterize Skeletal Muscle from Growth-Restricted Fetal Sheep. Am. J. Physiol.-Regul. Integr. Comp. Physiol..

[10] Bianco-Miotto T., Mayne B.T., Buckberry S., Breen J., Rodriguez L.C., Roberts C.T. (2016). Recent Progress Towards Understanding the Role of Dna Methylation in Human Placental Development. Reproduction.

[11] Robinson W.P., Price E.M. (2015). The Human Placental Methylome. Cold Spring Harb. Perspect. Med..

[12] Rahnama F., Shafiei F., Gluckman P.D., Mitchell M.D., Lobie P.E. (2006). Epigenetic Regulation of Human Trophoblastic Cell Migration and Invasion. Endocrinology.

[13] Serman L., Vlahović M., Sijan M., Bulić-Jakus F., Serman A., Sincić N., Matijević R., Jurić-Lekić G., Katusić A. (2007). The Impact of 5-Azacytidine on Placental Weight, Glycoprotein Pattern and Proliferating Cell Nuclear Antigen Expression in Rat Placenta. Placenta.

[14] Tan B., Zhou C., Zang X., Zhao X., Xiao L., Zeng J., Hong L., Wu Z., Gu T. (2023). Integrated Analysis of Dna Methylation and Gene Expression in Porcine Placental Development. Int. J. Mol. Sci..

[15] Peng W., Chen Y., Luo X., Shan N., Lan X., Olson D., Zhang H., Ding Y.B., Qi H.B. (2016). Dna Methylation-Associated Repression of Mest/Peg1 Expression Contributes to the Invasion of Extravillous Trophoblast Cells. Placenta.

[16] Ghazi T., Naidoo P., Naidoo R.N., Chuturgoon A.A. (2021). Prenatal Air Pollution Exposure and Placental Dna Methylation Changes: Implications on Fetal Development and Future Disease Susceptibility. Cells.

[17] King J.H., Kwan S., Yan J., Jiang X., Fomin V.G., Levine S.P., Wei E., Roberson M.S., Caudill M.A. (2019). Maternal Choline Supplementation Modulates Placental Markers of Inflammation, Angiogenesis, and Apoptosis in a Mouse Model of Placental Insufficiency. Nutrients.

[18] Grzeszczak K., Banocha-Arendarczyk N., Malinowski W., Ziętek P., Kosik-Bogacka D. (2023). Oxidative Stress in Pregnancy. Biomolecules.

[19] Chen Y.C., Sheen J.M., Tiao M.M., Tain Y.L., Huang L.T. (2013). Roles of Melatonin in Fetal Programming in Compromised Pregnancies. Int. J. Mol. Sci..

[20] Mark P.J., Crew R.C., Wharfe M.D., Waddell B.J. (2017). Rhythmic Three-Part Harmony: The Complex Interaction of Maternal, Placental and Fetal Circadian Systems. J. Biol. Rhythm..

[21] Voiculescu S.E., Zygouropoulos N., Zahiu C.D., Zagrean A.M. (2014). Role of Melatonin in Embryo Fetal Development. J. Med. Life.

[22] Ejaz H., Figaro J.K., Woolner A., Thottakam B., Galley H.F. (2020). Maternal Serum Melatonin Increases During Pregnancy and Falls Immediately After Delivery Implicating the Placenta as a Major Source of Melatonin. Front. Endocrinol..

[23] Berbets A.M., Davydenko I.S., Barbe A.M., Konkov D.H., Albota O.M., Yuzko O.M. (2021). Melatonin 1a and 1B Receptors’ Expression Decreases in the Placenta of Women with Fetal Growth Restriction. Reprod. Sci..

[24] Berbets A.M., Barbe A.M., Andriiets O.A., Andriiets A.V., Yuzko O.M. (2020). Melatonin Levels Decrease in the Umbilical Cord in Case of Intrauterine Growth Restriction. J. Med. Life.

[25] Wang L., Yan L., Han Q., Li G., Wu H., Ma X., Zhao M., Ma W., Ji P., Zhang R. (2023). Melatonin Supplementation During the Late Gestational Stage Enhances Reproductive Performance of Sows by Regulating Fluid Shear Stress and Improving Placental Antioxidant Capacity. Antioxidants.

[26] Korkmaz A., Reiter R.J. (2008). Epigenetic Regulation: A New Research Area for Melatonin?. J. Pineal Res..

[27] Qu J., Sun M., Wang X., Song X., He H., Huan Y. (2020). Melatonin Enhances the Development of Porcine Cloned Embryos by Improving Dna Methylation Reprogramming. Cell. Reprogram..

[28] Zhao S., Dong Y., Li Y., Wang Z., Chen Y., Dong Y. (2024). Melatonin Alleviates Lipopolysaccharide-Induced Abnormal Pregnancy through Mtnr1B Regulation of M6a. Int. J. Mol. Sci..

[29] Wu H., Zhou W., Liu H., Cui X., Ma W., Wu H., Li G., Wang L., Zhang J., Zhang X. (2022). Whole-Genome Methylation Analysis Reveals Epigenetic Variation Between Wild-Type and Nontransgenic Cloned, Asmt Transgenic Cloned Dairy Goats Generated by the Somatic Cell Nuclear Transfer. J. Anim. Sci. Biotechnol..

[30] Huang S., Wu Z., Huang Z., Hao X., Zhang L., Hu C., Wei J., Deng J., Tan C. (2021). Maternal Supply of Cysteamine Alleviates Oxidative Stress and Enhances Angiogenesis in Porcine Placenta. J. Anim. Sci. Biotechnol..

[31] Argente J., Mehls O., Barrios V. (2010). Growth and Body Composition in Very Young Sga Children. Pediatr. Nephrol..

[32] Maccani M.A., Marsit C.J. (2009). Epigenetics in the Placenta. Am. J. Reprod. Immunol..

[33] Flinn T., Kleemann D.O., Swinbourne A.M., Kelly J.M., Weaver A.C., Walker S.K., Gatford K.L., Kind K.L., van Wettere W. (2020). Neonatal Lamb Mortality: Major Risk Factors and the Potential Ameliorative Role of Melatonin. J. Anim. Sci. Biotechnol..

[34] Wang L., Han Q., Yan L., Ma X., Li G., Wu H., Liu Y., Chen H., Ji P., Wang B. (2024). Mtnr1B Deletion Disrupts Placental Angiogenesis through the Vegf Signaling Pathway Leading to Fetal Growth Restriction. Pharmacol. Res..

[35] Tan D.X., Hardeland R. (2021). The Reserve/Maximum Capacity of Melatonin’s Synthetic Function for the Potential Dimorphism of Melatonin Production and its Biological Significance in Mammals. Molecules.

[36] Soliman A., Lacasse A.A., Lanoix D., Sagrillo-Fagundes L., Boulard V., Vaillancourt C. (2015). Placental Melatonin System is Present Throughout Pregnancy and Regulates Villous Trophoblast Differentiation. J. Pineal Res..

[37] Wu J.J., Zheng X., Wu C., Ma W., Wang Y., Wang J., Wei Y., Zeng X., Zhang S., Guan W. (2025). Melatonin Alleviates High Temperature Exposure Induced Fetal Growth Restriction Via the Gut-Placenta-Fetus Axis in Pregnant Mice. J. Adv. Res..

[38] Chuffa L., Lupi L.A., Cucielo M.S., Silveira H.S., Reiter R.J., Seiva F. (2019). Melatonin Promotes Uterine and Placental Health: Potential Molecular Mechanisms. Int. J. Mol. Sci..

[39] Zhang X., Yun Y., Lai Z., Ji S., Yu G., Xie Z., Zhang H., Zhong X., Wang T., Zhang L. (2023). Supplemental Clostridium Butyricum Modulates Lipid Metabolism by Reshaping the Gut Microbiota Composition and Bile Acid Profile in Iugr Suckling Piglets. J. Anim. Sci. Biotechnol..

[40] Peng X., Cai X., Li J., Huang Y., Liu H., He J., Fang Z., Feng B., Tang J., Lin Y. (2021). Effects of Melatonin Supplementation During Pregnancy on Reproductive Performance, Maternal-Placental-Fetal Redox Status, and Placental Mitochondrial Function in a Sow Model. Antioxidants.

[41] Shao X., Yang Y., Liu Y., Wang Y., Zhao Y., Yu X., Liu J., Li Y.X., Wang Y.L. (2023). Orchestrated Feedback Regulation Between Melatonin and Sex Hormones Involving Gper1-Pka-Creb Signaling in the Placenta. J. Pineal Res..

[42] Tan B., Xiao L., Wang Y., Zhou C., Huang H., Li Z., Hong L., Cai G., Wu Z., Gu T. (2024). Comprehensive Analysis of Placental Dna Methylation Changes and Fetal Birth Weight in Pigs. Int. J. Mol. Sci..

[43] Linowiecka K., Slominski A.T., Reiter R.J., Böhm M., Steinbrink K., Paus R., Kleszczyński K. (2023). Melatonin: A Potential Regulator of Dna Methylation. Antioxidants.

[44] Li J., Deng Q., Fan W., Zeng Q., He H., Huang F. (2020). Melatonin-Induced Suppression of Dna Methylation Promotes Odontogenic Differentiation in Human Dental Pulp Cells. Bioengineered.

[45] Franczyk M., Lopucki M., Stachowicz N., Morawska D., Kankofer M. (2017). Extracellular Matrix Proteins in Healthy and Retained Placentas, Comparing Hemochorial and Synepitheliochorial Placentas. Placenta.

[46] Krause B.J., Carrasco-Wong I., Caniuguir A., Carvajal J., Farías M., Casanello P. (2013). Endothelial Enos/Arginase Imbalance Contributes to Vascular Dysfunction in Iugr Umbilical and Placental Vessels. Placenta.

[47] Kulandavelu S., Whiteley K.J., Bainbridge S.A., Qu D., Adamson S.L. (2013). Endothelial No Synthase Augments Fetoplacental Blood Flow, Placental Vascularization, and Fetal Growth in Mice. Hypertension.

[48] Sundrani D.P., Karkhanis A.R., Joshi S.R. (2021). Peroxisome Proliferator-Activated Receptors (Ppar), Fatty Acids and Micrornas: Implications in Women Delivering Low Birth Weight Babies. Syst. Biol. Reprod. Med..

[49] Aye I.L., Rosario F.J., Powell T.L., Jansson T. (2015). Adiponectin Supplementation in Pregnant Mice Prevents the Adverse Effects of Maternal Obesity on Placental Function and Fetal Growth. Proc. Natl. Acad. Sci. USA.

[50] Zhong J., Li J., Burton G.J., Koistinen H., Cheung K.W., Ng E., Yao Y., Yeung W., Lee C.L., Chiu P. (2024). The Functional Roles of Protein Glycosylation in Human Maternal-Fetal Crosstalk. Hum. Reprod. Update.

[51] Han K., Wang F., Yue Y., Tan X., Tian M., Miao Y., Zhao S., Dong W., Yu M. (2022). Glycomics Reveal that St6Gal1-Mediated Sialylation Regulates Uterine Lumen Closure During Implantation. Cell Proliferat..

[52] Abeln M., Albers I., Peters-Bernard U., Flächsig-Schulz K., Kats E., Kispert A., Tomlinson S., Gerardy-Schahn R., Münster-Kühnel A., Weinhold B. (2019). Sialic Acid is a Critical Fetal Defense Against Maternal Complement Attack. J. Clin. Investig..

