# Supplementary material for: Melatonin Supplementation During Middle Gestation Alleviates Intrauterine Growth Restriction in Piglets and Is Associated with Alterations in Placental DNA Methylation
Source: Int J Mol Sci. 2026 Oct 12;27(20):9046. doi: 10.3390/ijms27209046 (PMC13704602; doi:10.3390/ijms27209046)
Supplement: Supplementary file 1 [file ijms-27-09046-s001.zip › ijms-4568301-supplementary.pdf]

Melatonin Supplementation During Middle Gestation Alleviates Intrauterine Growth Restriction in Piglets and Is Associated with Alterations in Placental DNA Methylation

Likai Wang[1, 2 †], Huigang Han[1, 3 †], Zhenhua Xue[1], Laiqing Yan[1], Zixia Shen[1], Jiarui Gao[1], Pengyun Ji[1], Bingyuan Wang[1], Guoshi Liu[1, *]

[1] National Engineering Laboratory for Animal Breeding, Key Laboratory of Animal Genetics and Breeding of the Ministry of Agricultural, State Key Laboratory of Animal Biotech Breeding, Frontiers Science Center for Molecular Design Breeding, College of Animal Science and Technology, China Agricultural University, Beijing 100193, China

[2] Institutes of Biomedical Sciences, College of Life Science, Shanxi University, Taiyuan 030006, China

[3] College of Animal Science, Xinjiang Agricultural University, Urumq 830052, China

[*] Correspondence: gshliu@cau.edu.cn; Tel.: +86-18911383059

[†] These authors contributed equally to this work.

Supplementary Figure S1

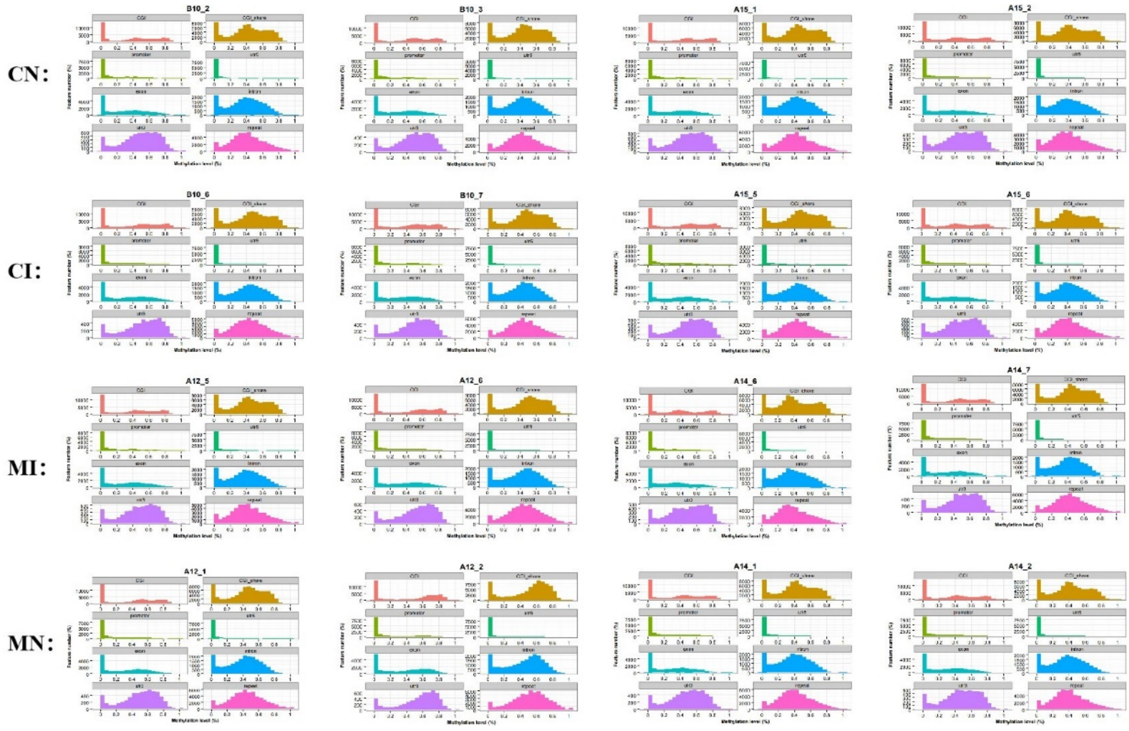

Figure S1. Number distribution of genomic elements under different methylation levels in samples. CN = CON group with normal birth weight piglets, CI = CON group with IUGR piglets, MN = LMT group with normal birth weight piglets, MI = LMT group with IUGR piglets, n = 4 per group. The horizontal coordinate represents the different methylation levels, and the vertical coordinate represents the number of functional elements at the corresponding methylation level. The overall methylation level of each functional region of each gene was the sum of the methylation levels of CpG sites in the region.

Supplementary Figure S2

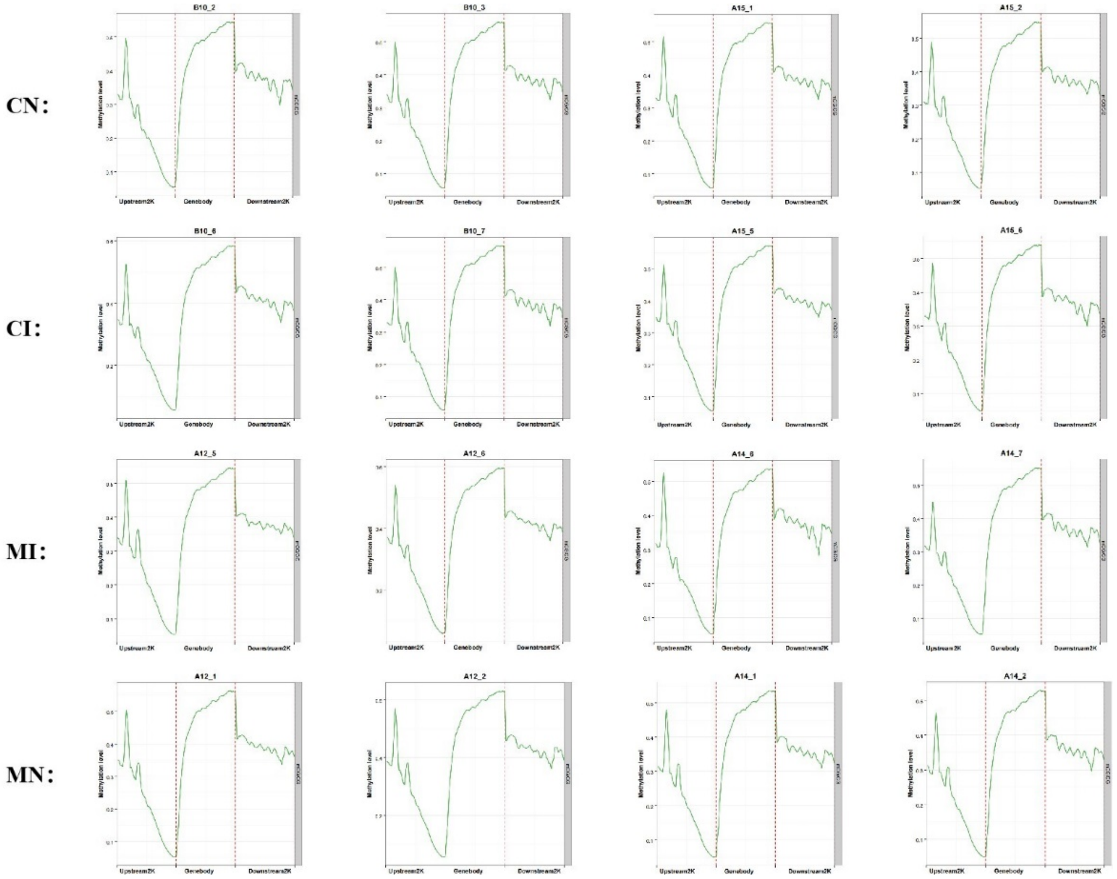

Figure S2. Distribution of sample methylation levels in the upstream and downstream 2K of genebody. The horizontal coordinates represent different regions, and the vertical coordinates represent methylation levels. Each region of each gene was equally divided into 50 bin, and then the corresponding bin C locus level of all regions was averaged. CN = CON group with normal birth weight piglets, CI = CON group with IUGR piglets, MN= LMT group with normal birth weight piglets, MI = LMT group with IUGR piglets, n = 4 per group.

Supplementary Figure S3

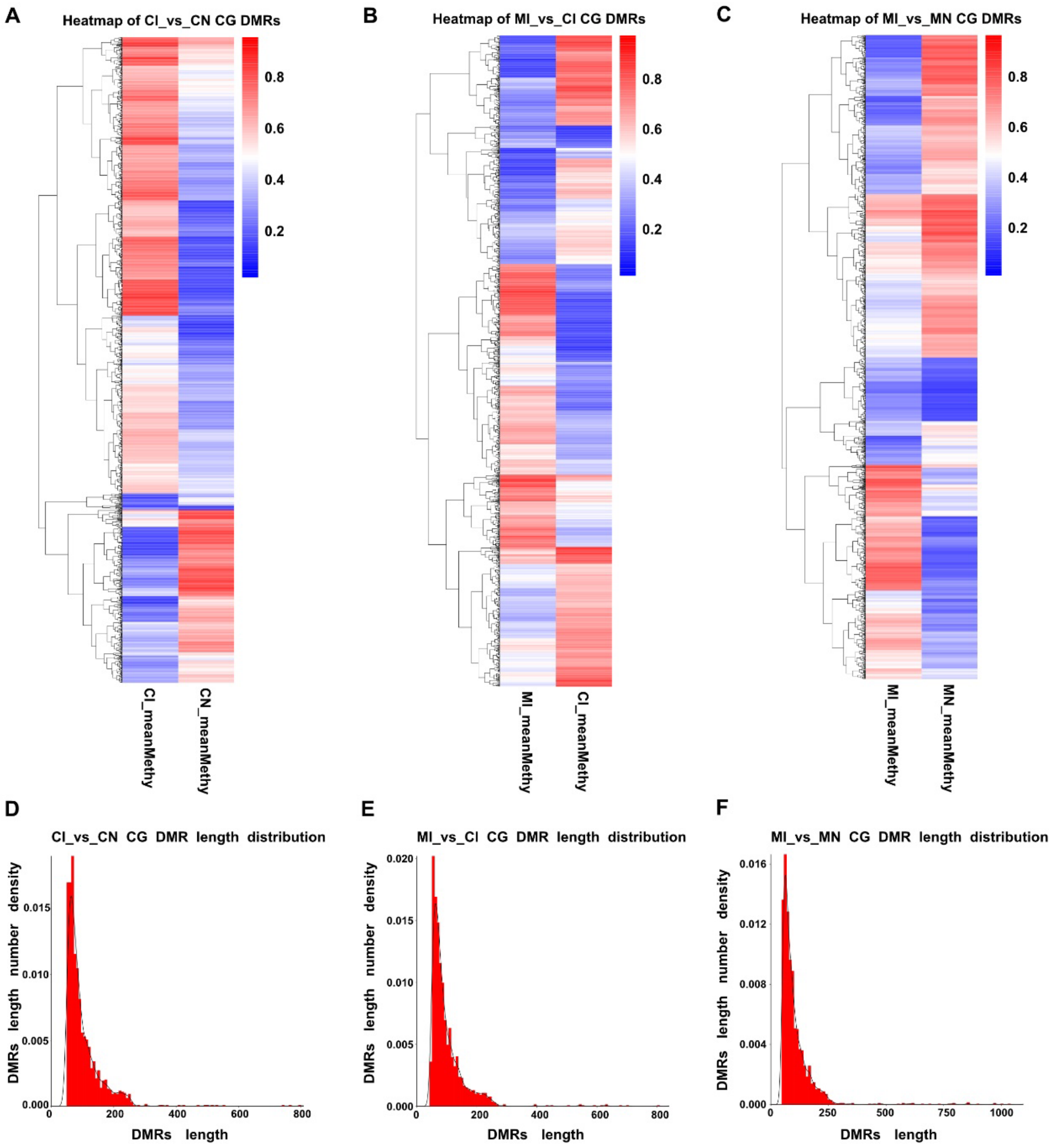

Figure S3. DMR methylation level clustering heat map and DMR length distribution. (A-C) DMR methylation level clustering heat map between different groups. (D-F) DMR length distribution among different groups. CN = CON group with normal birth weight piglets, CI = CON group with IUGR piglets, MN = LMT group with normal birth weight piglets, MI = LMT group with IUGR piglets, n = 4 per group.

Supplementary Table S1

The main primers of q-PCR

Gene	Sequences (Forward)	Sequences (Reverse)
Pig-*COL21A1*	ACTGTTTACCACAACCAGCGTC	CCGATTTGGGTTTGTCCG
Pig-*COL5A1*	GATGGCAAGTGGCACAGAGTC	GGTGGTCCGAGACAAAGAGC
Pig-*PPARA*	GGCTGCTATCATTTGGTGCG	TCGGGGTGGTTGGTCTGC
Pig-*ITPR3*	TGCCTGTTCAAGGTCTGCC	CGTGTCGTTCTGCTTCTGCT
Pig-*CACNA1S*	CTGGTCCGAACAGCACTCAA	GCCGATACCCATAATACTCCTCC
Pig-*PRKCA*	CCAACAACCTTGACCGAGTG	CAGTTCTTCCGTTCCCTTCC
Pig-*ST3GAL1*	CAACTCGGGCAACCTGAAG	CTCGGGGTACACGAAATGG
Pig-*GAPDH*	GTCGGTTGTGGATCTGACCTGC	GTCCTCAGTGTAGCCCAGGATG
